# Ball-Catching System Using Image Processing and an Omni-Directional Wheeled Mobile Robot

**DOI:** 10.3390/s21093208

**Published:** 2021-05-05

**Authors:** Sho-Tsung Kao, Ming-Tzu Ho

**Affiliations:** Department of Engineering Science, National Cheng Kung University, Tainan 701401, Taiwan; n98971035@gs.ncku.edu.tw

**Keywords:** visual servo, omni-directional mobile robot, Kalman filter, deep learning applications

## Abstract

The ball-catching system examined in this research, which was composed of an omni-directional wheeled mobile robot and an image processing system that included a dynamic stereo vision camera and a static camera, was used to capture a thrown ball. The thrown ball was tracked by the dynamic stereo vision camera, and the omni-directional wheeled mobile robot was navigated through the static camera. A Kalman filter with deep learning was used to decrease the visual measurement noises and to estimate the ball’s position and velocity. The ball’s future trajectory and landing point was predicted by estimating its position and velocity. Feedback linearization was used to linearize the omni-directional wheeled mobile robot model and was then combined with a proportional-integral-derivative (PID) controller. The visual tracking algorithm was initially simulated numerically, and then the performance of the designed system was verified experimentally. We verified that the designed system was able to precisely catch a thrown ball.

## 1. Introduction

The method in which visual information in a feedback control loop is used to precisely control the motion, position, and posture of a robot is called visual servoing. Visual servoing is well-known and commonly used in both dynamic and unstructured environments. Visual servoing tasks involving the use of a robot to catch a flying ball present a challenge.

In robotic ball-catching, there are several variations in the system configurations, methods of implementation, trajectory prediction algorithms, and control laws. In [1], the stereo camera system (two PAL cameras) was placed above the work area to catch a flying ball. In [2], two cameras were placed at the top and left of the work area for ping-pong ball catching and ball juggling. In [3], the cameras were located behind the robot for catching in-flight objects with uneven shapes. In [4], a high-speed vision system with two cameras was used for a ball juggling system.

In the above research, fixed cameras were used to predict the trajectory of the target object in the work area. The advantage is that the background image was fixed, and the image processing was relatively simple; however, it is difficult to achieve the desired results in an open space due to the field of view (FoV) constraints associated with the camera system and the limited workspace of a robotic arm.

In [5], an autonomous wheelchair mounted with a robotic arm used two vision sensors to accurately determine the location of the target object and to pick up the object. In [6], a robotic system endowed with only a single camera mounted in eye-in-hand configuration was used for the ball catching task. In [7], a robot with an eye-in-hand monocular camera was used for autonomous object grasping. The methods of [5,6,7] can resolve the stated problem. The eye-in-hand concept enables the camera to maneuver along with the robotic arm, which improves the trajectory prediction precision in an open space, especially when the object is near the robot. Another method by which to catch a ball in a wide-open space is to combine a static stereo vision system with a mobile robot to complete the ball-catching task, as described below.

In [8], the ball was tracked using two ceiling-mounted cameras and a camera mounted on the base of the robot. Each camera performed ball detection independently. The 3D coordinates of the ball were then triangulated from the 2D image locations. In [9], a robotic system consisted of a high-speed hand-arm, a high-speed vision system, and a real-time control system. A method was proposed to estimate the 3D position and orientation by fusing the information observed by a vision system and the contact information observed by tactile sensors.

In [10], the robot maintained a camera fixation that was centered on the image of the ball and kept the tangent of the camera angle rising at a constant rate. The performance advantage was principally due to the higher gain and effectively wider viewing angle when the camera remained centered on the ball image. In [11], a movable humanoid robot with an active stereo vision camera was designed and implemented for a ball-catching task. Two flying balls were caught by mobile humanoid robots at the same time in a wide space.

The prediction of the trajectory is an important factor for the effective capture of a flying ball. Most methods assumed that a free-flying object’s dynamic model was known. A parabola in 3D space was used to model the trajectory of a flying ball, and the least-squares method was used to estimate the model parameters.

In [10], the flying ball trajectory was predicted using a non-linear dynamic model that included air resistance with different parameter estimation algorithms. In [6], an estimate of the catching point was initially provided through a linear algorithm. Then, additional visual measurements were acquired to constantly refine the current estimate by exploiting a nonlinear optimization algorithm and a more accurate ballistic model with the influence of air resistance, visual measurement errors, and rotation caused by ground friction. One of the typical uses of the Kalman filter is in navigation and positioning technology.

In [12], an automated guided vehicle was combined with a Kalman filter with deep learning. The system was found to have good adaptability to the statistical properties of the noise system, which improved the positioning accuracy and prevented filter divergence. In our previous work [13], we provided a brief overview of an effective ball-catching system with an omni-directional wheeled mobile robot. In this paper, we extend the ball trajectory estimation method to improve the system’s ball-catching performance. Furthermore, the experiments and application are described in detail.

**Figure 1:**
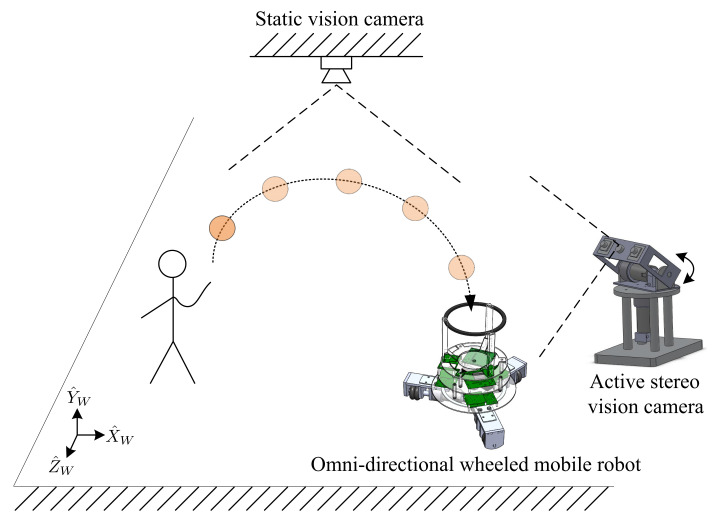
Schematic overview of the proposed system.

In this research, we developed a combined omni-directional wheeled mobile robot and a multi-camera vision system to catch a flying ball in a large workspace. Figure 1 provides a schematic diagram of the proposed system.

To maneuver the robot around, an omni-directional wheeled setup was selected due to its superiority in terms of mobility. This robot can enable translational or rotational movements or any combination of these two movements. An active stereo vision camera assumed the visual tracking task for the flying ball with two pan-and-tilt cameras. To navigate the mobile robot to the ball’s touchdown point, a static vision camera was used. Real-time processing of the image processing algorithms and control laws are necessary to accomplish these visual servoing tasks.

Therefore, digital signal processors (DSPs) were used to ensure the real-time ability to carry out these actions. Noise from the environment or other sources, such as the measurement of vision systems for caught balls, can deteriorate the performance of the proposed visual servoing system. In this work, estimating the position and velocity of the ball was achieved using the Kalman filter and a linear dynamic model of a flying ball. The use of deep learning in Kalman filtering improved both the accuracy and robustness of the results. In this paper, the experimental setup is presented, and the results of the simulation and experiments are provided to demonstrate the performance of the proposed system.

The remainder of this paper is composed as follows: In Section 2, the relationship between the coordinates used in this work is described (specifically, the world and image coordinates). In addition, the image processing algorithms are also introduced. Section 3 describes the active stereo vision camera. Section 4 describes the prediction and trajectory estimation method for a flying ball. Section 5 offers a discussion of the control law of the omni-directional wheeled mobile robot. Section 6 presents the implementation of the designed system. Section 7 presents the results for the simulation and the experiments. Finally, Section 8 provides our concluding remarks.

## 2. Image Processing and Visual Measurement

Vision systems are used to obtain the location of the ball and robot in a three-dimensional (3D) Cartesian coordinate system. The position determination of an object from an image is based on a pinhole camera model of the vision system [14,15]. The position of an object can be given in the vector form as

(1) $$\lambda p=K_{I}\left[R_{E}|T\right]P_{o}$$

where vector $p={\left[x_{im}\;y_{im}\;1\right]}^{T}$ represents the 2D homogeneous coordinates of an image point in the image coordinate system. The vector $P_{o}={\left[X_{W}\;Y_{W}\;Z_{W}\;1\right]}^{T}$ represents the 3D homogeneous coordinate of a target object point in the world coordinate system. The 3 by 3 matrix $R_{E}$ and 3D vector *T* are external camera parameters that define the rotation and translation between the world frame and camera frame, respectively. λ is a scaling factor, and $K_{I}$ is the internal parameter matrix of the camera. It is given by

(2) $$K_{I}=\left[\begin{array}{ccc} f_{sx} & f_{s\theta } & o_{x}\\ 0 & f_{sy} & o_{y}\\ 0 & 0 & 1 \end{array}\right].$$

In the matrix $K_{I}$, $\left(o_{x},o_{y}\right)$ is the principal point in the image coordinate system in pixels; $f_{sx}$ and $f_{sy}$ are the size of the unit lengths in the horizontal and vertical pixels, respectively, and $f_{s\theta }$ is the skewness factor of the pixels. The camera calibration procedure in [14] is used to calibrate the internal camera parameters beforehand.

**Figure 2:**
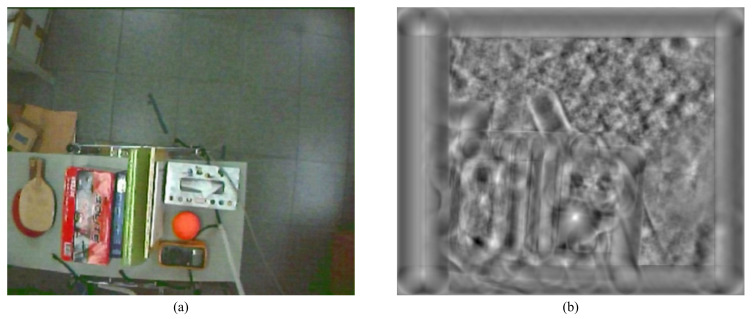
(**a**) Image with a complicated background captured by the camera and (**b**) the normalized cross-correlation image.

In the image plane, to obtain the precise position of a target object, a series of image processing algorithms are used to process the images captured from the camera. The source image with a complex background acquired from the camera is shown in Figure 2a. In this study, the template matching approach [16] is used to find the image of the target object that matches a template object image in the whole image. The template matching process compares the sub-image of the source image and template object image, from left to right and from top to bottom, to obtain the correlation between these two images.

Additionally, to detect the target object in the three-dimensional space, the template object image is resized during the comparison process. Figure 2b shows the normalized cross-correlation image, in which the brightest one represents the most similarity between two images.

**Figure 3:**
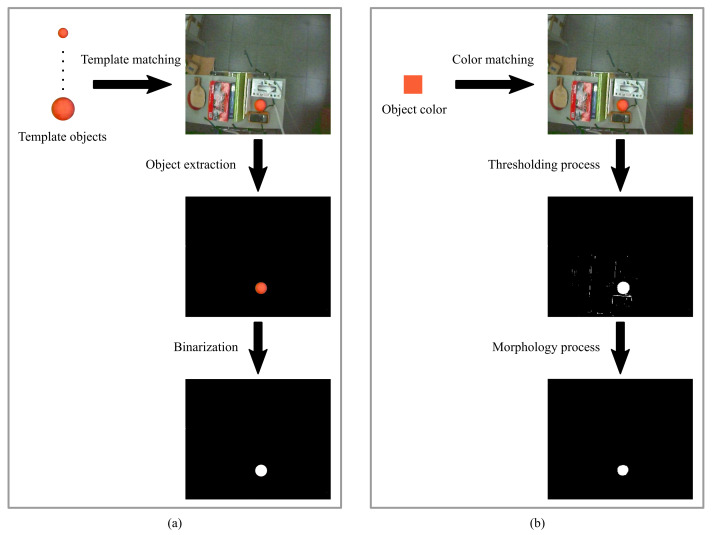
Simulation results of the (**a**) template matching method and (**b**) color matching method.

The simulation result of the template matching was compared to the one obtained by the color matching method. The result when using the template matching method to perform ball detection is shown in Figure 3a. The result based on the color matching method is shown in Figure 3b. In a complex background, when comparing the object extraction step in Figure 3a with the thresholding process in Figure 3b, the use of template matching for ball detection is not interfered with by other objects, which is suitable for the environment used in this study.

The location of the processed image was taken using the centroid of the object as follows:

(3) $$\left(x_{c},y_{c}\right)=\left(\frac{1}{n_{1}}\sum x_{im},\frac{1}{n_{1}}\sum y_{im}\right)$$

where $\left(x_{c},y_{c}\right)$ are the center coordinates of the object, $\left(x_{im},y_{im}\right)$ are the coordinates of the white pixel, and $n_{1}$ is the number of white pixels. The actual centroid of the ball in Figure 3 obtained by the manual image segmentation is $\left(x_{c},y_{c}\right)=\left(336,368\right)$. The centroid of the ball obtained from the template matching method is $\left(x_{c},y_{c}\right)=\left(334,365\right)$, and the centroid of the ball obtained from the color matching method is $\left(x_{c},y_{c}\right)=\left(322,355\right)$. The template matching method provided a more accurate result.

## 3. Active Stereo Vision

**Figure 4:**
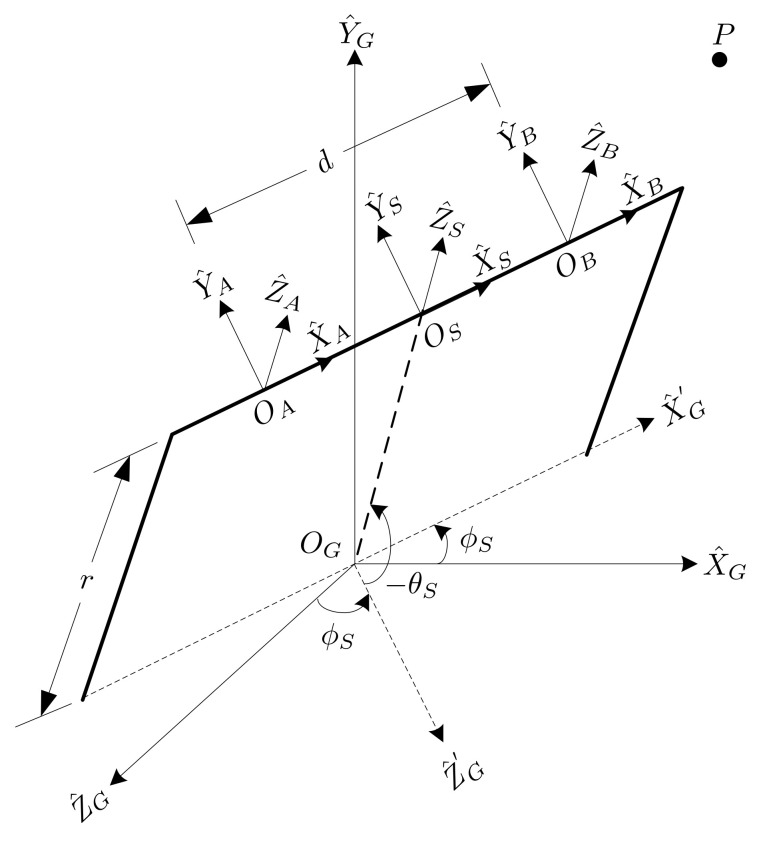
Coordinate frames.

In this study, an active stereo vision camera was used to locate and trace the flying ball. The active stereo vision camera included two cameras mounted on a pan-and-tilt platform, and the cameras were parallel to each other. The coordinate systems of this active vision camera are described in Figure 4, for which the parameters are listed below.

$$\begin{array}{cc} P & \;\mathrm{target}\;\mathrm{object}\;\mathrm{in}\;\mathrm{the}\;\mathrm{word}\;\mathrm{coordinate}\;\mathrm{system}.\\ r & \;\mathrm{the}\;\mathrm{distance}\;\mathrm{between}\;\mathrm{the}\;\mathrm{stereo}\;\mathrm{coordinate}\;\mathrm{and}\;\mathrm{the}\;\mathrm{geodetic}\;\mathrm{coordinate}.\\ d & \;\mathrm{the}\;\mathrm{distance}\;\mathrm{between}\;\mathrm{the}\;\mathrm{center}\;\mathrm{of}\;\mathrm{camera}\;A\;\mathrm{and}\;\mathrm{camera}\;B.\\ \phi_{s} & \;\mathrm{the}\;\mathrm{angle}\;\mathrm{of}\;\mathrm{the}\;\mathrm{pan}\;\mathrm{axis}.\\ \theta_{s} & \;\mathrm{the}\;\mathrm{angle}\;\mathrm{of}\;\mathrm{the}\;\mathrm{tilt}\;\mathrm{axis}.\\ \left(O_{A},{\hat{X}}_{A},{\hat{Y}}_{A},{\hat{Z}}_{A}\right) & \;\mathrm{coordinate}\;\mathrm{system}\;\mathrm{of}\;\mathrm{camera}\;A.\\ \left(O_{B},{\hat{X}}_{B},{\hat{Y}}_{B},{\hat{Z}}_{B}\right) & \;\mathrm{coordinate}\;\mathrm{system}\;\mathrm{of}\;\mathrm{camera}\;B.\\ \left(O_{S},{\hat{X}}_{S},{\hat{Y}}_{S},{\hat{Z}}_{S}\right) & \mathrm{stereo}\;\mathrm{coordinate}\;\mathrm{system}\;\mathrm{located}\;\mathrm{in}\;\mathrm{the}\;\mathrm{center}\;\mathrm{of}\;\mathrm{the}\;\mathrm{camera}\;\mathrm{frame}\;A\;\mathrm{and}\;\mathrm{camera}\;\mathrm{frame}\;B\;\mathrm{to}\;\mathrm{point}\;\mathrm{to}\;\mathrm{the}\;\mathrm{target}.\\ \left(O_{G},{\hat{X}}_{G},{\hat{Y}}_{G},{\hat{Z}}_{G}\right) & \mathrm{geodetic}\;\mathrm{coordinate}\;\mathrm{system}\;\mathrm{fixed}\;\mathrm{relative}\;\mathrm{to}\;\mathrm{the}\;\mathrm{ground}\;\mathrm{with}\;\mathrm{the}\;\mathrm{pan}\;\mathrm{axis}\;\mathrm{aligned}\;\mathrm{on}\;\mathrm{the}{\hat{Y}}_{G}-\mathrm{axis}\;\mathrm{and}\;\mathrm{the}\;\mathrm{tilt}\;\mathrm{axis}\;\mathrm{aligned}\;\mathrm{on}\;\mathrm{the}{\hat{X}}_{G}-\mathrm{axis},\;\mathrm{where}\;\mathrm{both}\;\mathrm{axes}\;\mathrm{are}\;\mathrm{connected}\;\mathrm{and}\;\mathrm{considered}\;\mathrm{to}\;\mathrm{be}\;\mathrm{zero}.\;\mathrm{Three}-\mathrm{axes}\;\mathrm{are}\;\mathrm{determined}\;\mathrm{using}\;a\;\mathrm{right}-\mathrm{handed}\;\mathrm{coordinate}\;\mathrm{system}. \end{array}$$

The pan angle $\phi_{S}$ rotates along the ${\hat{Y}}_{G}$ axis, and the tilt angle $\theta_{S}$ rotates along the ${\hat{X}}_{G}^{{}^{\prime }}$ axis. The position vector of the target object *P* relative to camera A is denoted as ${\left[x_{A}\;y_{A}\;z_{A}\right]}^{T}$. In this study, we assumed that cameras A and B were identical. Based on (1), after obtaining the image coordinates of the objects of camera A and camera B, respectively, the position of the target object in the coordinate frame of camera A can be written as:

(4) $$\begin{array}{ccc} x_{A} & = & \frac{d}{x_{A\_im}-x_{B\_im}}[x_{A\_im}-o_{x}-\frac{f_{s\theta }}{f_{sy}}\left(y_{A\_im}-o_{y})\right]\\ y_{A} & = & d\frac{f_{sx}}{f_{sy}}\;\cdot \;\frac{y_{A\_im}-o_{y}}{x_{A\_im}-x_{B\_im}}\\ z_{A} & = & f_{sx}\frac{d}{x_{A\_im}-x_{B\_im}} \end{array}$$

where $\left(x_{A\_im},y_{A\_im}\right)$ and $\left(x_{B\_im},y_{B\_im}\right)$ are the coordinates of the target object obtained from cameras A and B, respectively. According to Figure 4, the relationship of the coordinate system between the stereo coordinate and the camera A coordinate can be given by:

(5) $$\left[\begin{array}{c} x_{S}\\ y_{S}\\ z_{S} \end{array}\right]=\left[\begin{array}{c} x_{A}\\ y_{A}\\ z_{A} \end{array}\right]-\left[\begin{array}{c} d/2\\ 0\\ 0 \end{array}\right]$$

where ${\left[x_{S}\;y_{S}\;z_{S}\right]}^{T}$ is the position vector of target object *P* relative to the stereo coordinate frame. The position vector of target object *P* converts to the geodetic frame using a homogeneous transformation matrix $H_{S}^{G}$, which is given by:

(6) $$\left[\begin{array}{c} x_{G}\\ y_{G}\\ z_{G}\\ 1 \end{array}\right]=H_{S}^{G}\left[\begin{array}{c} x_{S}\\ y_{S}\\ z_{S}\\ 1 \end{array}\right]$$

where ${\left[x_{G}\;y_{G}\;z_{G}\right]}^{T}$ is the position of target object *P* relative to the geodetic coordinate system and

$$H_{S}^{G}=\left[\begin{array}{cccc} \cos\phi_{s} & -\sin\phi_{s}\sin\theta_{s} & \sin\phi_{s}\cos\theta_{s} & r\sin\phi_{s}\cos\theta_{s}\\ 0 & \cos\theta_{s} & \sin\theta_{s} & r\sin\theta_{s}\\ -\sin\phi_{s} & -\cos\phi_{s}\sin\theta_{s} & \cos\phi_{s}\cos\theta_{s} & r\cos\phi_{s}\cos\theta_{s}\\ 0 & 0 & 0 & 1 \end{array}\right].$$

Using Equations (4)–(6), the pixel position of the target object in the image frames of the two cameras can be used to determine the position vector of target object *P* in the world frame.

**Figure 5:**
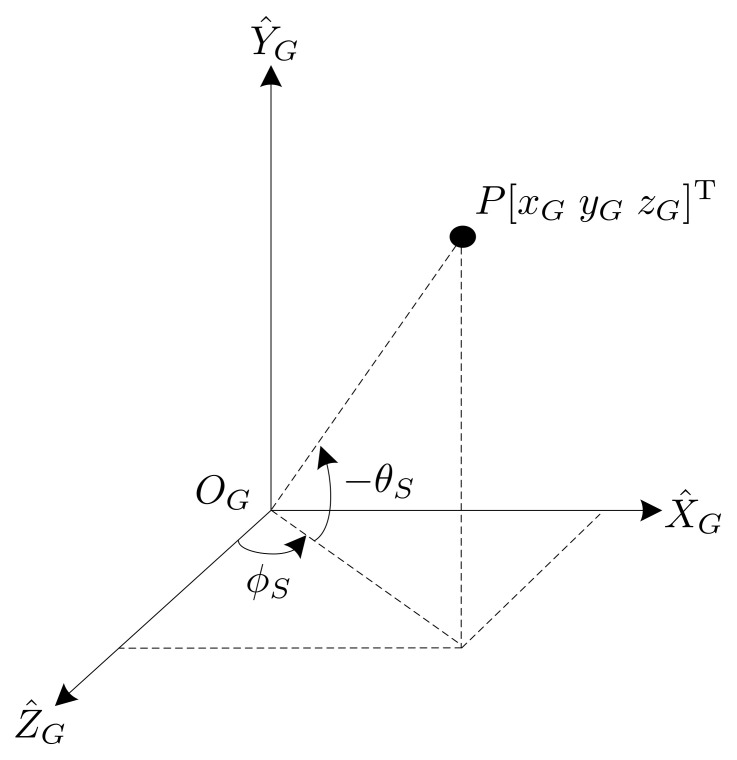
Angular motor displacements.

Figure 5 illustrates the position of target object *P* in the geodetic frame. From Figure 5 using simple geometry, the angular displacement of the pan-axis can be determined by

(7) $$\phi_{S}=\sin^{-1}\frac{x_{G}}{\sqrt{x_{G}^{2}+z_{G}^{2}}}$$

where the angular displacement of the tilt-axis is:

(8) $$\theta_{S}=\sin^{-1}\frac{y_{G}}{\sqrt{x_{G}^{2}+y_{G}^{2}+z_{G}^{2}}}$$

The direct current (DC) servo motors drive the pan-and-tilt platform to maintain the continuous tracking of $\theta_{S}$ and $\phi_{S}$. The $\theta_{S}$ and $\phi_{S}$ angular commands are sent in real-time to keep the target object in the FoV of the cameras.

## 4. Trajectory Estimation and Prediction

Visual tracking is challenging due to image variations caused by camera noise, background changes, illumination changes, and object occlusion. The above-mentioned problems will deteriorate the tracking performance and may even cause the loss of target objects. In this work, a Kalman filter [17,18] was applied to enhance the robustness of the designed visual tracking system for the purpose of estimating the target object’s position and velocity. The projectile motion trajectory for the flying ball was used to predict the touchdown point. The mobile robot was commanded to catch the ball at the appropriate location. A brief introduction to the Kalman filter is given below.

A state-space system model is described by

(9) $$\begin{array}{cc} x_{k+1}= & Ax_{k}+Bu_{k}+\varepsilon_{k}\\ y_{k}= & Cx_{k}+\omega_{k} \end{array}$$

where $x_{k}$ is the state of the system; $u_{k}$ is the input, and $y_{k}$ is the measurement. Matrices *A*, *B*, and *C* are the state transition matrix, input matrix, and output matrix, respectively. The state and measurement noises are denoted as $\varepsilon_{k}$ and $\omega_{k}$. The zero-mean normal-distribution Gaussian white noise is assumed to be these two noises, and the covariances are $Q_{k}$ and $R_{k}$, respectively.

The use of the Kalman filter involves two major procedures: the time updating step and the measurement updating step [17,18]. Time updating is used to estimate the probability outcome of the next state. The measurement updating step is used to update the estimated state with the measured information. This updated state is used in the time updating step of the next cycle. The Kalman filter is done recursively, and the recursive formulas are

- Time updating step:
  (10) $$\begin{array}{cc} {\hat{x}}_{k}^{-}= & A{\hat{x}}_{k-1}+Bu_{k-1}\\ P_{k}^{-}= & AP_{k-1}A^{T}+Q_{k}. \end{array}$$
- Measurement updating step:
  (11) $$\begin{array}{ccc} K_{k} & = & P_{k}^{-}C^{T}{\left(CP_{k}^{-}C^{T}+R_{k}\right)}^{-1} \end{array}$$
  (12) $$\begin{array}{ccc} {\hat{x}}_{k} & = & {\hat{x}}_{k}^{-}+K_{k}\left(y_{k}-C{\hat{x}}_{k}^{-}\right) \end{array}$$
  (13) $$\begin{array}{ccc} P_{k} & = & \left(I-K_{k}C\right)P_{k}^{-}. \end{array}$$

In (10)–(13), ${\hat{x}}_{k}^{-}$ denotes the a priori predicted state, and ${\hat{x}}_{k}$ is the optimal estimated state after the measurement is updated. $P_{k}^{-}$ and $P_{k}$ are the a priori and a posteriori estimate error covariances, respectively. $K_{k}$ is known as the Kalman gain, and $y_{k}-{\hat{y}}_{k}$ is called the measurement residual. Based on (11), the a posteriori estimate error covariance $P_{k}$ is minimized by $K_{k}$, and ${\hat{x}}_{k}$ is, hence, optimized.

Now, we consider the dynamics of a flying ball. The position and velocity vector of the ball are denoted as ${\left[x_{W}\;y_{W}\;z_{W}\right]}^{T}$ and ${\left[v_{x}\;v_{y}\;v_{z}\right]}^{T}$ in the world coordinate system, respectively. We assumed that the flying trajectory of the ball is not interfered with by other objects. The forces considered in this work are the gravitational force, the buoyancy force, and the drag force. Other non-stated forces, such as the Magnus force [19], are ignored.

The buoyancy force vector is denoted as ${\left[0\;\;0\;\;\frac{4}{3}\pi R^{3}\rho g\right]}^{T}$, where the radius of the ball is R(m); the air density is $\rho \;(\mathrm{kg}/m^{3})$, and the gravitational acceleration at sea level is $g\;(m/s^{2})$. The drag force is assumed to be proportional to the velocity. According to Stokes’s law [19], the drag force is $6\pi \mu Rv_{w}$, where μ(kg/m·s) is the dynamic viscosity of the air, and $v_{w}={\left[v_{x}\;v_{y}\;v_{z}\right]}^{T}$. Let the mass of the ball be denoted as m(kg). The equation that governs the motion of a flying ball can be written as

(14) $$m\frac{dv_{w}}{dt}=\left[\begin{array}{c} 0\\ -m^{\prime }g\\ 0 \end{array}\right]-bv_{w}$$

where

(15) $$\begin{array}{ccc} b & ≜ & 6\pi \mu R \end{array}$$

(16) $$\begin{array}{ccc} m^{\prime } & ≜ & m-\frac{4}{3}\pi R^{3}\rho . \end{array}$$

By discretizing (14) with the sampling period Δt, we obtain the dynamic model of the system as shown below:

(17) $$\begin{array}{ccc} x_{W}\left(t\right) & = & v_{x}\left(t-\Delta t\right)\Delta t+x_{W}\left(t-\Delta t\right) \end{array}$$

(18) $$\begin{array}{ccc} y_{W}\left(t\right) & = & v_{y}\left(t-\Delta t\right)\Delta t+y_{W}\left(t-\Delta t\right) \end{array}$$

(19) $$\begin{array}{ccc} z_{W}\left(t\right) & = & v_{z}\left(t-\Delta t\right)\Delta t+z_{W}\left(t-\Delta t\right) \end{array}$$

(20) $$\begin{array}{ccc} v_{x}\left(t\right) & = & \left(1-\frac{b}{m}\right)v_{x}\left(t-\Delta t\right)\Delta t \end{array}$$

(21) $$\begin{array}{ccc} v_{y}\left(t\right) & = & -\frac{m^{\prime }}{m}g\Delta t+\left(1-\frac{b}{m}\right)v_{y}\left(t-\Delta t\right)\Delta t \end{array}$$

(22) $$\begin{array}{ccc} v_{z}\left(t\right) & = & \left(1-\frac{b}{m}\right)v_{z}\left(t-\Delta t\right)\Delta t. \end{array}$$

From Equations (17)–(22), a discrete-time linear state-space form (9) can be further written for the system model as

(23) $$\begin{array}{cc} x_{k+1}= & {\left[\begin{array}{cccccc} x_{W}\left(t\right) & y_{W}\left(t\right) & z_{W}\left(t\right) & v_{x}\left(t\right) & v_{y}\left(t\right) & v_{z}\left(t\right) \end{array}\right]}^{T}\\ x_{k}= & [\begin{array}{ccc} x_{W}\left(t-\Delta t\right) & y_{W}\left(t-\Delta t\right) & z_{W}\left(t-\Delta t\right) \end{array}\\ \; & \begin{array}{ccc} v_{x}\left(t-\Delta t\right) & v_{y}\left(t-\Delta t\right) & v_{z}\left(t-\Delta t\right) \end{array}{]}^{T}\\ u_{k}= & -g \end{array}$$

$$\begin{array}{c} A=\left[\begin{array}{cccccc} 1 & 0 & 0 & \Delta t & 0 & 0\\ 0 & 1 & 0 & 0 & \Delta t & 0\\ 0 & 0 & 1 & 0 & 0 & \Delta t\\ 0 & 0 & 0 & (1-\frac{b}{m})\Delta t & 0 & 0\\ 0 & 0 & 0 & 0 & (1-\frac{b}{m})\Delta t & 0\\ 0 & 0 & 0 & 0 & 0 & (1-\frac{b}{m})\Delta t \end{array}\right] \end{array}$$

$$\begin{array}{c} B={\left[\begin{array}{cccccc} 0 & 0 & 0 & 0 & \frac{m^{\prime }}{m}\Delta t & 0 \end{array}\right]}^{T} \end{array}$$

$$\begin{array}{c} C=\left[\begin{array}{cccccc} 1 & 0 & 0 & 0 & 0 & 0\\ 0 & 1 & 0 & 0 & 0 & 0\\ 0 & 0 & 1 & 0 & 0 & 0\\ 0 & 0 & 0 & 1 & 0 & 0\\ 0 & 0 & 0 & 0 & 1 & 0\\ 0 & 0 & 0 & 0 & 0 & 1 \end{array}\right]. \end{array}$$

Based on the above model, the Kalman filter is applicable for visual tracking to estimate the ball’s trajectory. Therefore, for the visual tracking of the flying ball, the Kalman filter and a constant acceleration model were used in this work.

Using the estimated position, the estimated velocity, and the projectile motion Formulas (17)–(22), the future trajectory and touchdown point were predicted for the flying ball. Since the uncertainty of the initial value $P_{0}$ affects the estimation accuracy and convergence of the Kalman filter, this can cause the active stereo vision camera to fail to track the ball, and therefore the ball leaves the FoV. The inaccuracy of the initial value is the result of an unreasonable flight trajectory, such as the ball not being thrown into the air.

**Figure 6:**
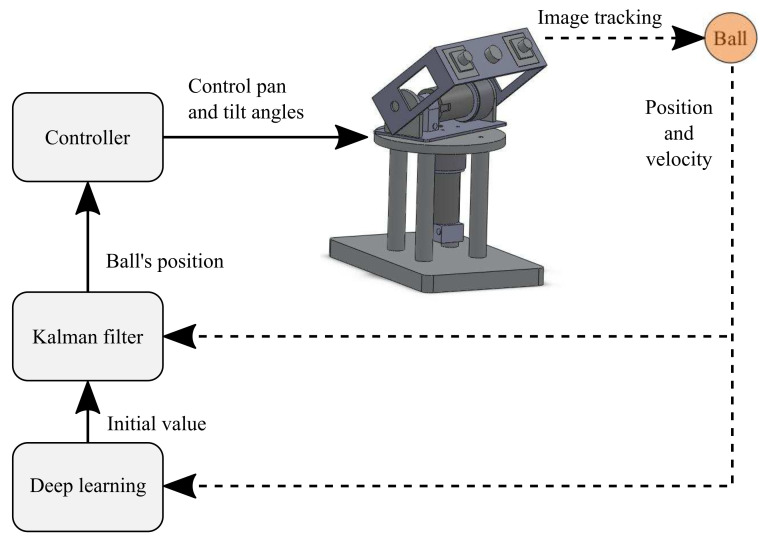
Kalman filter combined with deep learning.

In this work, deep learning was used to obtain the initial value of the state estimation error covariance $P_{0}$. Figure 6 describes the application using the Kalman filter combined with deep learning. After the camera obtains the position and velocity of the flying ball, the Kalman filter estimates the next position and commands the active stereo vision camera to track the ball.

**Figure 7:**
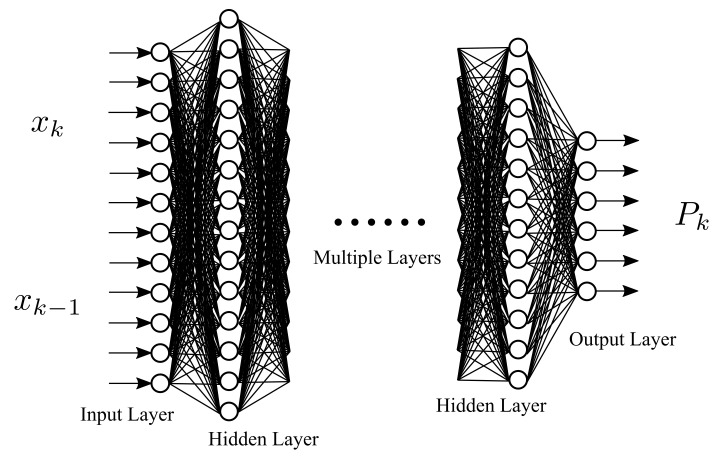
The architecture of the deep learning.

Figure 7 describes the deep learning architecture. The input layer receives the input $x_{k}$ and $x_{k-1}$, which is the data learned by the neural network. The network is based on AlexNet [20], which is a deep convolutional neural network. These input matrices represent the position vector and velocity vector of the flying ball. The last layer is called the output layer, which outputs an initial estimated error covariance $P_{0}$, representing the neural network’s result. The hidden layers are performed in the layers between the input and output layers. Deep learning is helpful to attenuate the initial value deviation in the filtering process. Once the ball’s predicted touchdown point is obtained, the point-to-point path planning is used to command the mobile robot to move toward the touchdown point in advance to catch the ball. This ball-catching strategy is discussed in a later section.

## 5. Controller Design of the Omni-Directional Wheeled Mobile Robot

**Figure 8:**
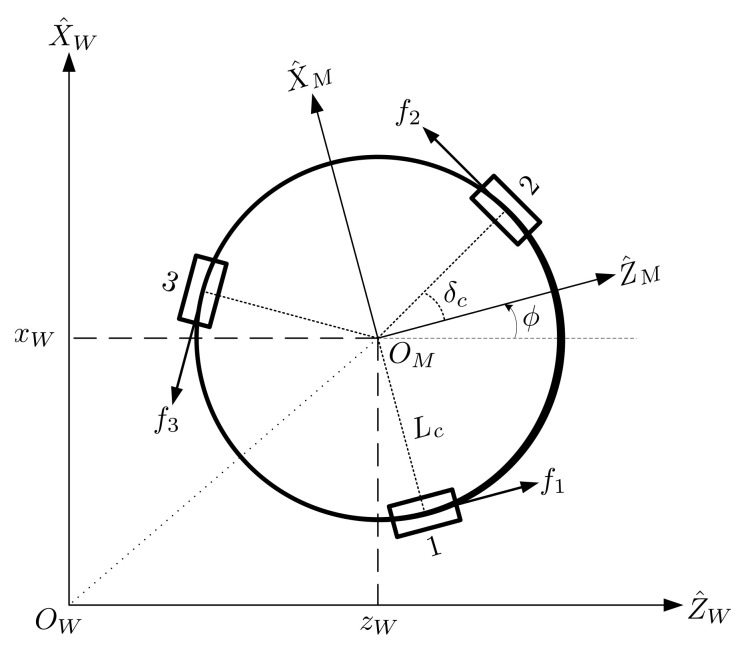
Top view of the omni-directional mobile robot.

Figure 8 shows a schematic diagram of an omni-directional wheeled mobile robot from the top view. This mobile robot consists of a rigid body and three omni-directional wheels labeled 1, 2, and 3. The wheels were arranged at an equal distance from the center of the robot platform with a $120^{\circ }$ interval. This setup allowed the robot to move freely in any direction. In this study, ($O_{W},{\hat{Z}}_{W},{\hat{X}}_{W}$) is the world coordinate system. ($O_{M},{\hat{Z}}_{M},{\hat{X}}_{M}$) is the body coordinate system with the origin attached to the center of mass of the robot. The direction of the ${\hat{X}}_{M}$-axis is aligned to wheel 1, as shown in Figure 8. We assumed that the wheels can roll without slipping.

The parameters for an omni-directional mobile robot are listed as follows:

$$\begin{array}{ccc} M_{t} & & \mathrm{mass}\;\mathrm{of}\;\mathrm{the}\;\mathrm{mobile}\;\mathrm{robot}.\\ L_{c} & & \mathrm{radius}\;\mathrm{of}\;\mathrm{the}\;\mathrm{mobile}\;\mathrm{robot}.\\ f_{1},f_{2},f_{3} & & \mathrm{the}\;\mathrm{reaction}\;\mathrm{force}\;\mathrm{applied}\;\mathrm{by}\;\mathrm{the}\;\mathrm{ground}\;\mathrm{to}\;\mathrm{the}\;\mathrm{omni}-\mathrm{directional}\;\mathrm{wheel},\;\mathrm{where}\;\mathrm{the}\;\mathrm{direction}\;\mathrm{is}\;\mathrm{vertical}\;\mathrm{to}\;\mathrm{wheel}\;\mathrm{axes}\;1,\;2,\;\mathrm{and}\;3,\;\mathrm{respectively}.\\ R_{w} & & \mathrm{radius}\;\mathrm{of}\;\mathrm{the}\;\mathrm{omni}-\mathrm{directional}\;\mathrm{wheels}.\\ \delta_{c} & & \mathrm{the}\;\mathrm{angle}\;\mathrm{between}\;\mathrm{wheel}\;2\;\mathrm{and}\;\mathrm{the}\;{\hat{Z}}_{M}-\mathrm{axis}\;\mathrm{of}\;\mathrm{the}\;\mathrm{mobile}\;\mathrm{coordinate}\;\mathrm{system};\;\mathrm{the}\;\mathrm{value}\;\mathrm{is}\;\mathrm{fixed}\;\mathrm{to}\;30^{\circ }.\\ \phi & & \mathrm{the}\;\mathrm{rotation}\;\mathrm{angle}\;\mathrm{of}\;\mathrm{the}\;\mathrm{omni}-\mathrm{directional}\;\mathrm{mobile}\;\mathrm{robot}.\\ I_{y} & & \mathrm{moment}\;\mathrm{of}\;\mathrm{inertia}\;\mathrm{of}\;\mathrm{the}\;\mathrm{omni}-\mathrm{directional}\;\mathrm{mobile}\;\mathrm{robot}\;\mathrm{about}\;{\hat{Y}}_{M}-\mathrm{axis}.\\ \theta_{1},\theta_{2},\theta_{3} & & \mathrm{the}\;\mathrm{rotation}\;\mathrm{angle}\;\mathrm{of}\;\mathrm{omni}-\mathrm{directional}\;\mathrm{wheels}\;1,\;2,\;\mathrm{and}\;3,\;\mathrm{respectively}.\\ \omega_{1},\omega_{2},\omega_{3} & & \mathrm{the}\;\mathrm{angular}\;\mathrm{velocity}\;\mathrm{of}\;\mathrm{omni}-\mathrm{directional}\;\mathrm{wheels}\;1,\;2,\;\mathrm{and}\;3,\;\mathrm{respectively}.\\ {\left[z_{W}\;x_{W}\right]}^{T} & & \mathrm{the}\;\mathrm{position}\;\mathrm{for}\;\mathrm{the}\;\mathrm{center}\;\mathrm{of}\;\mathrm{mass}\;\mathrm{of}\;\mathrm{the}\;\mathrm{robot}\;\mathrm{relative}\;\mathrm{to}\;\mathrm{the}\;\mathrm{world}\;\mathrm{coordinate}\;\mathrm{system}. \end{array}$$

According to [21], the dynamics and kinematics of the mobile robot can be described by:

(24) $$\left[\begin{array}{c} {\ddot{z}}_{W}\\ {\ddot{x}}_{W}\\ \ddot{\phi } \end{array}\right]=\left[\begin{array}{ccc} M_{t} & 0 & 0\\ 0 & M_{t} & 0\\ 0 & 0 & I_{y} \end{array}\right]\left[\begin{array}{ccc} \cos\phi & -\sin\phi & 0\\ \sin\phi & \cos\phi & 0\\ 0 & 0 & 1 \end{array}\right]\left[\begin{array}{ccc} 1 & -\frac{1}{2} & -\frac{1}{2}\\ 0 & \frac{\sqrt{3}}{2} & -\frac{\sqrt{3}}{2}\\ L_{c} & L_{c} & L_{c} \end{array}\right]\left[\begin{array}{c} f_{1}\\ f_{2}\\ f_{3} \end{array}\right]$$

(25) $$\left[\begin{array}{c} \omega_{1}\\ \omega_{2}\\ \omega_{3} \end{array}\right]=\frac{1}{R_{w}}\left[\begin{array}{ccc} 1 & 0 & L_{c}\\ -\frac{1}{2} & \frac{\sqrt{3}}{2} & L_{c}\\ -\frac{1}{2} & -\frac{\sqrt{3}}{2} & L_{c} \end{array}\right]\left[\begin{array}{ccc} \cos\phi & \sin\phi & 0\\ -\sin\phi & \cos\phi & 0\\ 0 & 0 & 1 \end{array}\right]\left[\begin{array}{c} {\dot{z}}_{W}\\ {\dot{x}}_{W}\\ \dot{\phi } \end{array}\right].$$

In this study, the brushed DC servo motors drove the omni-directional wheels. We also assumed that the motor’s electrical time constant was smaller than the mechanical time constants and that the motor friction was negligible. The model of a DC motor is, thus, reduced to

(26) $$\tau_{m}=\frac{K_{t}}{R_{a}}u-\frac{{K_{t}}^{2}}{R_{a}}\omega_{m}.$$

In (26), $\tau_{m}$, *u*, $K_{t}$, $\omega_{m}$, and $R_{a}$ represent the motor torque, control voltage, motor torque constant, angular velocity of the motor, and armature resistance, respectively. The traction force *f* of the wheel is given by

(27) $$f=\frac{n}{R_{w}}\tau_{m}$$

where *n* is the gear ratio. The three motors used in this mobile robot were assumed to be identical. Therefore, combining (26) and (27), the relationship between *f*, *u*, and ω is given by

(28) $$\left[\begin{array}{c} f_{1}\\ f_{2}\\ f_{3} \end{array}\right]=\frac{nK_{t}}{R_{w}R_{a}}\left[\begin{array}{c} u_{1}\\ u_{2}\\ u_{3} \end{array}\right]-\frac{n^{2}{K_{t}}^{2}}{R_{w}R_{a}}\left[\begin{array}{c} \omega_{1}\\ \omega_{2}\\ \omega_{3} \end{array}\right].$$

From (24), (25), and (28), the dynamics of the robot can be presented as

(29) $${\ddot{P}}_{w}=A_{w}{\dot{P}}_{w}+B_{w}\left(\phi \right)U_{C}$$

where

(30) $$P_{w}={\left[\begin{array}{ccc} z_{W} & x_{W} & \phi \end{array}\right]}^{T},U_{C}={\left[\begin{array}{ccc} u_{1} & u_{2} & u_{3} \end{array}\right]}^{T}$$

(31) $$A_{w}=\left[\begin{array}{ccc} a_{1} & 0 & 0\\ 0 & a_{1} & 0\\ 0 & 0 & a_{2} \end{array}\right]$$

(32) $$B_{w}\left(\phi \right)=\left[\begin{array}{cc} 2b_{1}\cos\left(\phi \right) & -b_{1}\cos\left(\phi \right)-\sqrt{3}b_{1}\sin\left(\phi \right)\\ 2b_{1}\sin\left(\phi \right) & -b_{1}\sin\left(\phi \right)+\sqrt{3}b_{1}\cos\left(\phi \right)\\ b_{2} & b_{2} \end{array}\right.\left.\begin{array}{cc} \; & -b_{1}\cos\left(\phi \right)+\sqrt{3}b_{1}\sin\left(\phi \right)\\ \; & -b_{1}\sin\left(\phi \right)-\sqrt{3}b_{1}\cos\left(\phi \right)\\ \; & b_{2} \end{array}\right]$$

with

(33) $$a_{1}=\frac{-3n^{2}K_{t}^{2}}{2R_{w}^{2}M_{t}R_{a}},a_{2}=\frac{-3n^{2}K_{t}^{2}L_{c}^{2}}{R_{w}^{2}I_{y}R_{a}},b_{1}=\frac{nK_{t}}{2R_{w}M_{t}R_{a}},b_{2}=\frac{nK_{t}L_{c}}{R_{w}I_{y}R_{a}}.$$

We assume that the predicted touchdown point of the ball in the world coordinate system is ${\left[z_{bw}\;x_{bw}\right]}^{T}$. The position reference to the mobile robot is set to be:

$$\begin{array}{c} P_{bw}\left(t\right)={\left[\begin{array}{ccc} z_{bw}\left(t\right) & x_{bw}\left(t\right) & \phi \end{array}\right]}^{T} \end{array}$$

where the rotation angle ϕ is assumed to be 0. The tracking error is defined as follows:

$$\begin{array}{c} e\left(t\right)=P_{bw}\left(t\right)-P_{w}\left(t\right). \end{array}$$

This gives

(34) $$\ddot{e}={\ddot{P}}_{bw}-\left[A_{w}{\dot{P}}_{w}+B_{w}\left(\phi \right)U_{C}\right].$$

A new control input *U* [22] is defined as follows:

(35) $$U≜{\ddot{P}}_{bw}-\left[A_{w}{\dot{P}}_{w}+B_{w}\left(\phi \right)U_{C}\right].$$

From (35) and (34), we obtain

(36) $$\frac{d}{dt}\left[\begin{array}{c} e\\ \dot{e} \end{array}\right]=\left[\begin{array}{cc} 0 & I\\ 0 & 0 \end{array}\right]\left[\begin{array}{c} e\\ \dot{e} \end{array}\right]+\left[\begin{array}{c} 0\\ I \end{array}\right]U.$$

From (34), the feedback control $U_{c}$ can be written as

$$\begin{array}{c} U_{C}=B_{w}{\left(\phi \right)}^{-1}\left[{\ddot{P}}_{bw}-A_{w}{\dot{P}}_{w}-U\right]. \end{array}$$

In the form used in (36), the system is decoupled into a linear system, and the PID control algorithm is used for tracking control. In this case, the following PID control:

(37) $$\begin{array}{cc} \dot{\varepsilon }= & e\\ U= & -K_{p}e-K_{d}\dot{e}-K_{i}\varepsilon \end{array}$$

where $K_{d}$, $K_{p}$, and $K_{i}$ are 3 by 3 diagonal PID gain matrixes that equal $\mathrm{diag}\{k_{d_{i}}\}$, $\mathrm{diag}\{k_{p_{i}}\}$, and $\mathrm{diag}\{k_{i_{i}}\}$, respectively, and *i* = 1, 2, and 3. From (36) and (37), it follows that the closed-loop tracking error system is given by:

$$\begin{array}{c} \frac{d}{dt}\left[\begin{array}{c} \varepsilon \\ e\\ \dot{e} \end{array}\right]=\left[\begin{array}{ccc} 0 & I & 0\\ 0 & 0 & I\\ -K_{i} & -K_{p} & -K_{d} \end{array}\right]\left[\begin{array}{c} \varepsilon \\ e\\ \dot{e} \end{array}\right]. \end{array}$$

According to the Routh–Hurwitz criterion [23], PID gain values $k_{p}$, $k_{i}$, and $k_{d}$ must satisfy:

$$\begin{array}{c} k_{i_{i}}<k_{d_{i}}k_{p_{i}},i=1,2,3. \end{array}$$

To obtain closed-loop stability, the PID control gain values are based on the control design method proposed in [24]. The phase margin and the gain margin were set to $45^{\circ }$ and 6.0206 dB in this work.

## 6. Implementation of the Designed System

**Figure 9:**
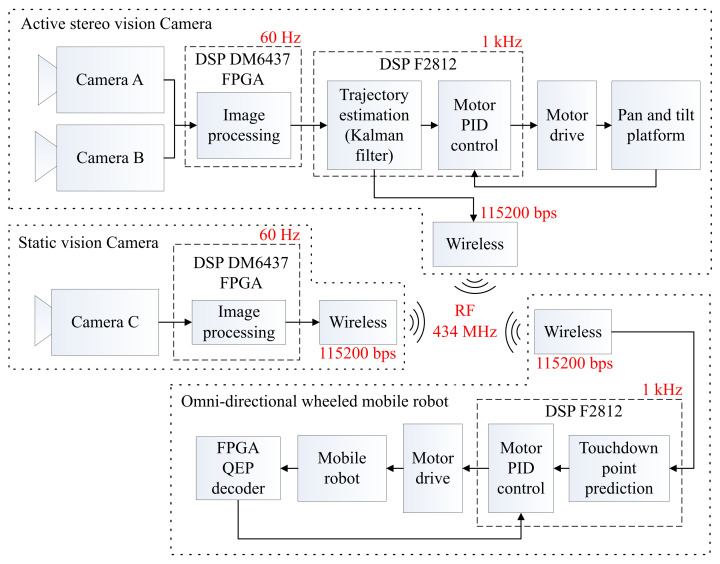
Block diagram of the proposed system.

Figure 9 shows a block diagram of the proposed ball-catching system. This system consisted of an omni-directional wheeled mobile robot and an image processing system that included an active stereo vision camera and a static vision camera.

**Figure 10:**
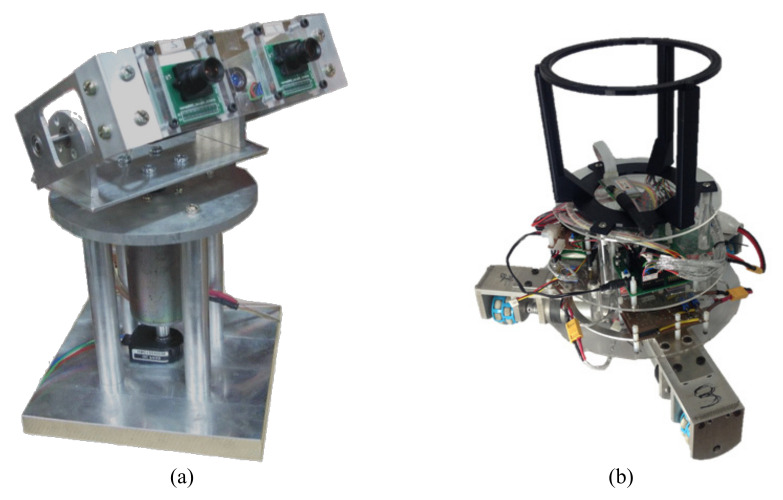
Omni-directional wheeled mobile robot.

Figure 10a shows the active stereo vision camera. The MT9P001 image sensor was used, which is a complementary metal-oxide-semiconductor (CMOS). It can capture 640 × 480 pixels in the quantized RGB format at 60 frames per second (FPS). The cameras were attached onto a field-programmable gate array (FPGA) board. This FPGA board was used to configure the cameras and to acquire images. In the real-time image process for the acquired images, a DSP (TMS320DM6437) board was used.

An optical encoder with the resolution of 500 pulses/rev was used to measure the motors’ angular displacement in the pan-and-tilt platform. Another DSP board (TMS320F2812), with two quadrature encoder pulse (QEP) units and one pulse width modulation (PWM) signal generator unit was used to acquire the angular displacement and rotational direction of the motor from the quadrature encoder and to control the pan-and-tilt platform motors. In addition, the Kalman filter was implemented to mitigate the measurement noises and to predict the motion of the ball.

The static vision camera was mounted above the work area, where the FoV of the camera covered the entire work area (length: 2.5 m, width: 2.5 m, and height: 3 m) as shown in Figure 1. This camera was used to locate and navigate the omni-directional wheeled mobile robot. As with the active stereo vision camera, the Kalman-filter-based vision tracking and image processing algorithms were implemented in the DSP board (TMS320DM6437).

The mobile robot, as stated in Section 6, consisted of three brushed DC motors used to drive the omni-directional wheels. A DSP board (TMS320F2812) was used for PID control of the motors and the touchdown point prediction for the ball. To obtain the wheel’s angular displacement, an optical encoder with a 500 pulse/rev resolution was mounted to the shaft of each wheel. These optical encoders generate quadrature encoder signals to the QEP circuit on an FPGA board for decoding. The wheels’ angular velocities were obtained by differentiating the angular displacement using the sampling time, and a low-pass filter was then applied to attenuate the high-frequency noises.

The robot’s position and orientation were determined with the static vision camera and a dead reckoning algorithm based on the motor encoder measurements. The PWM signals were generated according to the designed PID control laws to drive each of the motors. Figure 10b shows a basket 0.16 m in diameter mounted on the top of the robot for the purpose of catching the ball.

All of the systems stated above were communicated with using wireless communication modules, as shown in Figure 9. The active stereo vision camera obtained the position and velocity of the ball and then sent it to the mobile robot to predict the touchdown point. The static vision camera obtained the position and direction of the mobile robot, and then sent it to the mobile robot for navigation and positioning through wireless communication.

## 7. Simulation and Experimental Results

### 7.1. Touchdown Point Prediction

The prediction of the touchdown point of the target ball was first verified through a numerical simulation using MATLAB/Simulink. The mass and the radius of the ball were set at 0.07 kg and 0.004 m, respectively. The initial location of the ball in meters was ${\left[3.9\;\;\;1.1\;\;\;5.15\right]}^{T}$, and the initial velocity in meters per second was ${\left[-2.1\;\;\;5.2\;\;\;-1.9\right]}^{T}$. The sampling period was 0.0167 s. Although the dynamics of a flying ball were considered, it was impossible to precisely model the ball’s rotation and the airflow field conditions during flight.

$Q_{k}$ mainly represents these noises. Since these noises have little effect on the flight of the ball, $Q_{k}$ can be reasonably obtained according to experimental measurements. $R_{k}$ models the light variation noise, which cannot be processed by the camera calibration method; however, the slight variation was not significant in this study. A reasonable value of $R_{k}$ can be obtained through experimental measurements. Thus, the covariance matrices $Q_{k}$ and $R_{k}$ were assumed to be constant during the motion, and those used in this simulation are given below.

$$\begin{array}{c} Q_{k}=\left[\begin{array}{cccccc} 0.01 & 0 & 0 & 0 & 0 & 0\\ 0 & 0.01 & 0 & 0 & 0 & 0\\ 0 & 0 & 0.01 & 0 & 0 & 0\\ 0 & 0 & 0 & 0.005 & 0 & 0\\ 0 & 0 & 0 & 0 & 0.005 & 0\\ 0 & 0 & 0 & 0 & 0 & 0.005 \end{array}\right] \end{array}$$

$$\begin{array}{c} R_{k}=\left[\begin{array}{cccccc} 0.8 & 0 & 0 & 0 & 0 & 0\\ 0 & 0.8 & 0 & 0 & 0 & 0\\ 0 & 0 & 0.8 & 0 & 0 & 0\\ 0 & 0 & 0 & 0.16 & 0 & 0\\ 0 & 0 & 0 & 0 & 0.16 & 0\\ 0 & 0 & 0 & 0 & 0 & 0.16 \end{array}\right]. \end{array}$$

The initial error covariance matrix prediction was

$$\begin{array}{c} P_{0}=\left[\begin{array}{cccccc} 1.6\times 10^{-9} & 0 & 0 & 3.2\times 10^{-7} & 0 & 0\\ 0 & 1.6\times 10^{-9} & 0 & 0 & 3.2\times 10^{-7} & 0\\ 0 & 0 & 1.6\times 10^{-9} & 0 & 0 & 3.2\times 10^{-7}\\ 3.2\times 10^{-7} & 0 & 0 & 6.4\times 10^{-5} & 0 & 0\\ 0 & 3.2\times 10^{-7} & 0 & 0 & 6.4\times 10^{-5} & 0\\ 0 & 0 & 3.2\times 10^{-7} & 0 & 0 & 6.4\times 10^{-5} \end{array}\right]. \end{array}$$

**Figure 11:**
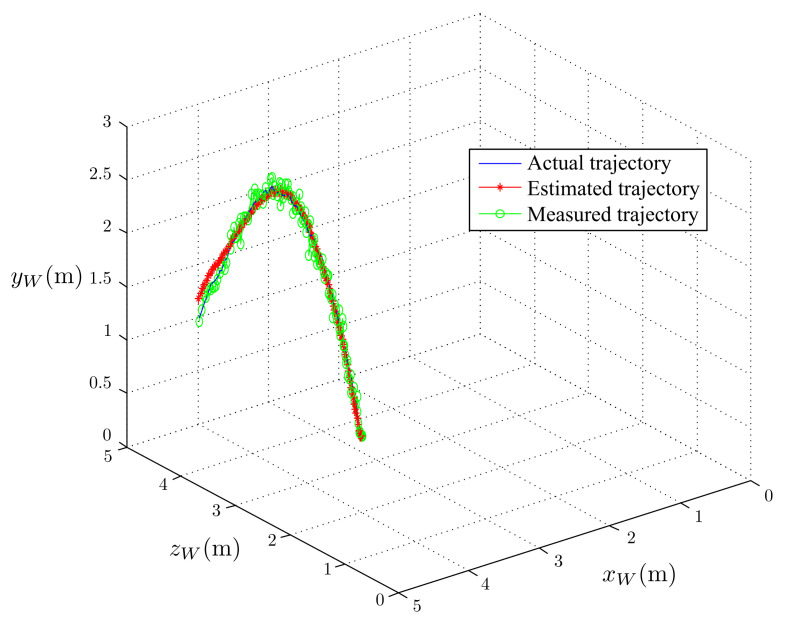
Comparison of the actual trajectory, estimated trajectory, and measured trajectory.

**Figure 12:**
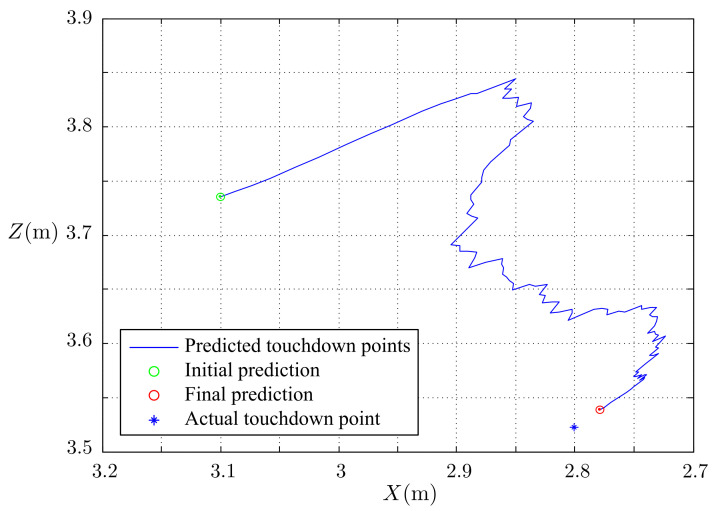
Prediction of the touchdown points.

The simulated results of the estimated, measured, and actual trajectories of the flying ball are shown in Figure 11. We observed that the initial estimated and actual trajectories were significantly different. After several iterations, the estimated trajectory overlapped the actual trajectory with reasonable accuracy. The predicted touchdown point obtained using Equations (17)–(22) in each step of the estimation is plotted in Figure 12. The initially predicted location was quite far from the actual one; however, it converged to the real touchdown point with additional iterations.

### 7.2. Improvement of Projectile Prediction

**Figure 13:**
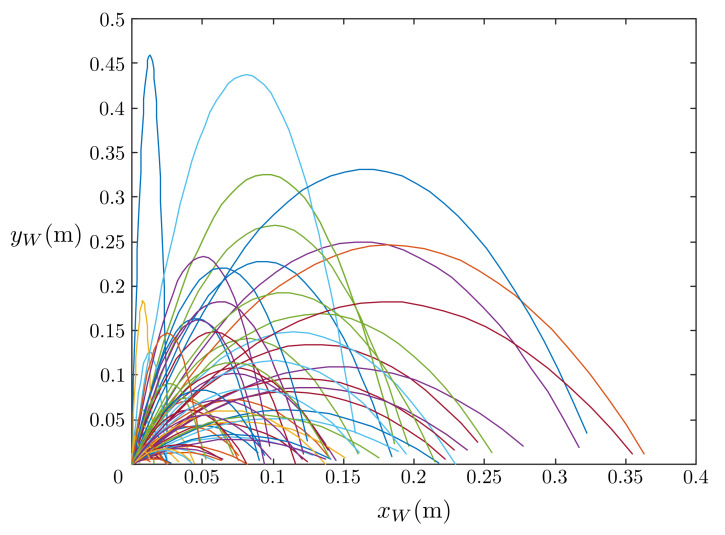
The training data sets.

**Figure 14:**
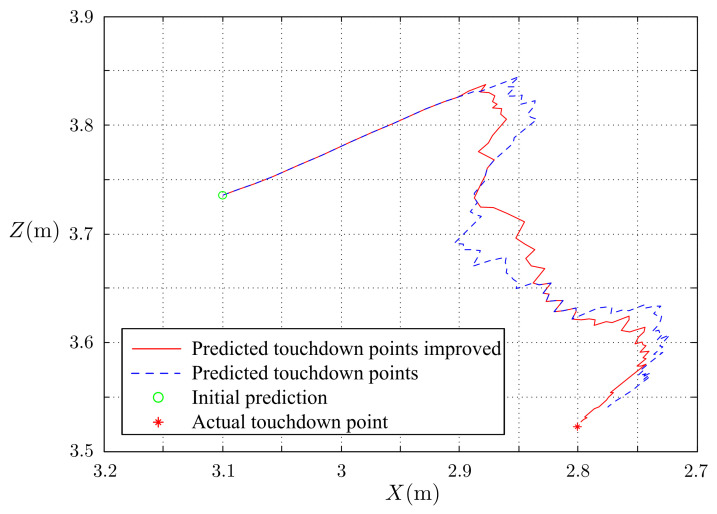
Prediction of the touchdown points improved by deep learning.

Deep learning was used to improve the projectile prediction. The training data sets are shown in Figure 13, with a total of 80 projectile data. The deep learning ANN was trained using many projectile trajectory data in Figure 13 to accurately obtain the ball’s projectile in the initial state to set a reasonable $P_{0}$ value. The covariance matrices $Q_{k}$ and $R_{k}$ were the same as shown in the above simulation, and the initial estimate error covariance matrix $P_{0}$ was obtained through the deep learning ANN after two iterations of measurements, and the Kalman filter was set to update $P_{k}$ during the ball motion. Figure 14 shows the result of predicting the touchdown point using $P_{0}$ obtained by the deep learning ANN. From the figure, the use of deep learning increased the accuracy of predicting the touchdown point.

### 7.3. Free Falling Ball Experimental

**Figure 15:**
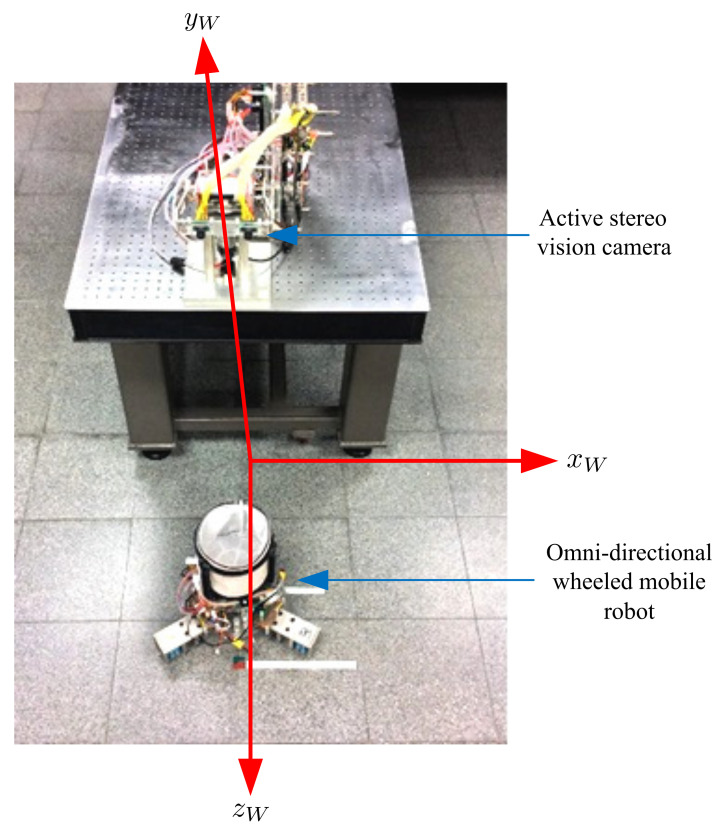
The experimental setup.

For further validation, the proposed visual servoing system for ball-catching was developed and performed in the experimental setup shown in Figure 15. An active stereo vision camera achieved the visual tracking task for the flying ball with two pan-and-tilt cameras. The omni-directional wheeled mobile robot was navigated to catch the ball.

**Figure 16:**
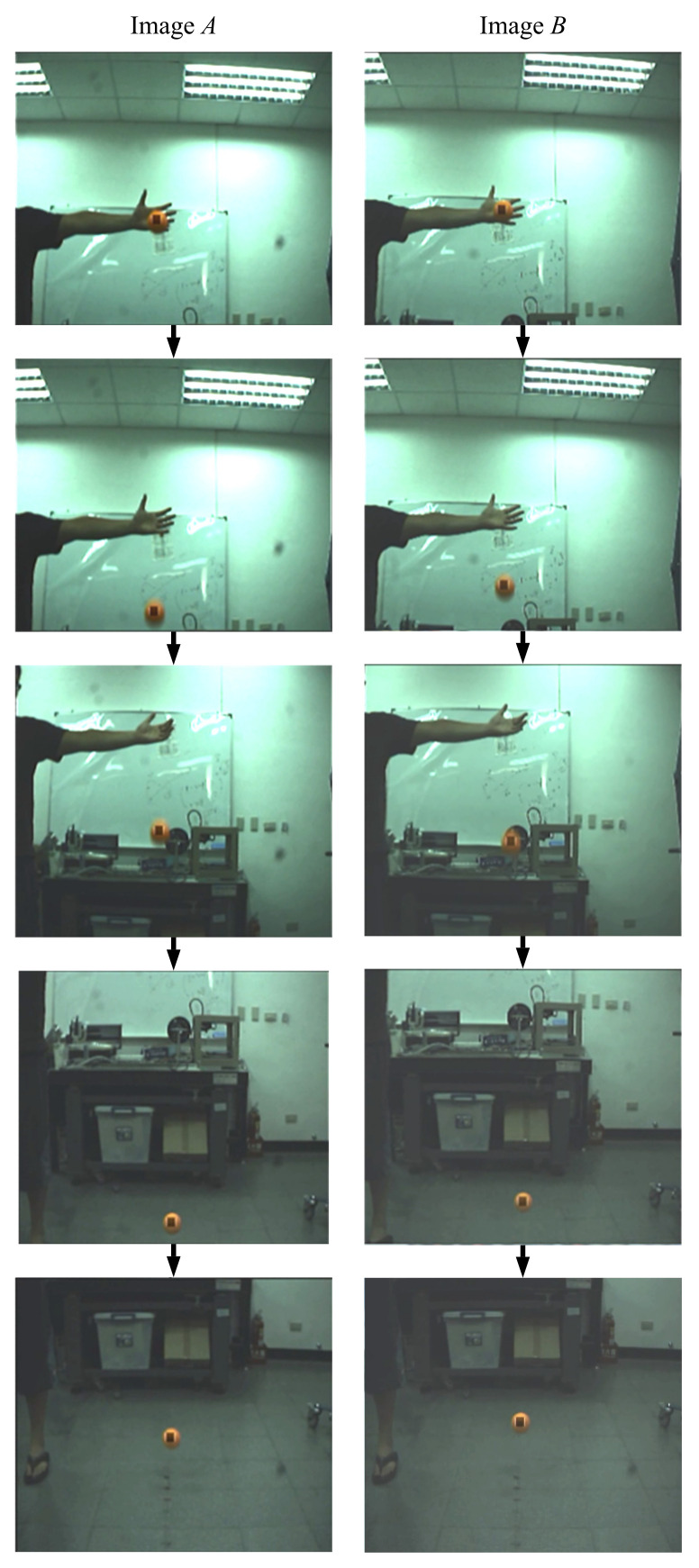
A free falling ball: the sequence of images captured.

**Figure 17:**
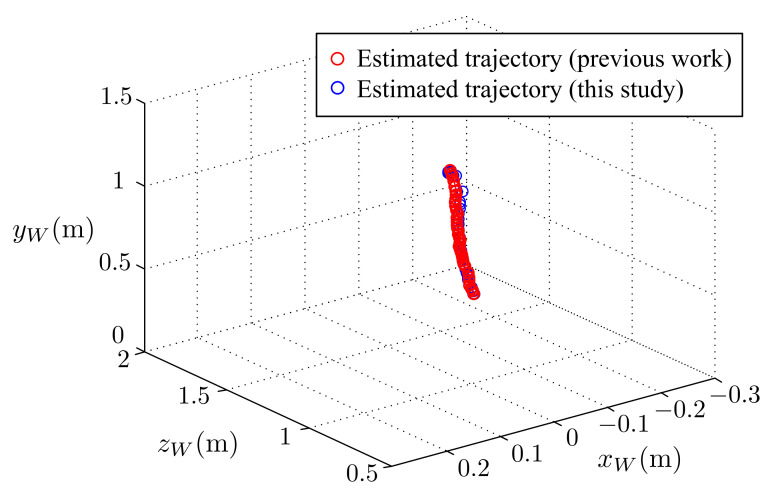
A free falling ball: estimated trajectory of the ball.

**Figure 18:**
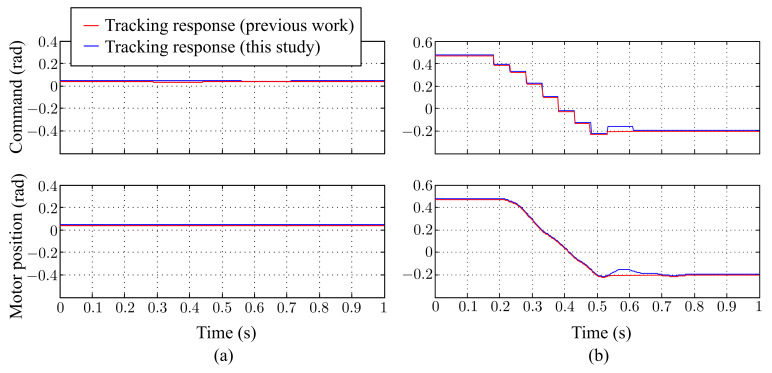
A free falling ball: tracking response of (**a**) the pan-axis motor and (**b**) the tilt-axis motor.

To verify the performance of the active stereo vision camera, an orange ball was dropped a suitable distance in front of the system. The visual tracking system was expected to be able to track the ball and keep it in the FoV of both cameras. Figure 16 shows a series of images taken by the active stereo vision camera. In both image series (image A and image B), the ball was kept in the images. The results of a free falling ball visual tracking of this study and the results of our previous work are given in the following Figure 17 and Figure 18.

Figure 17 shows the estimated trajectory of the ball, and Figure 18 shows the command and time response of the pan and tilt angles, respectively. In the case of a free-drop object, the object only moved in the vertical direction with no horizontal displacement. As shown in Figure 18a, the command and time response of the tilt angle changed over time, while the pan angle remained at 0 the entire time. Both the pan and tilt motor were able to precisely follow the commands. Figure 18b, compared with our previous results, shows that the tracking response was improved in this study. With these results, we concluded that the active stereo vision camera was able to successfully perform visual tracking.

### 7.4. Catching a Flying Ball Experiment

**Figure 19:**
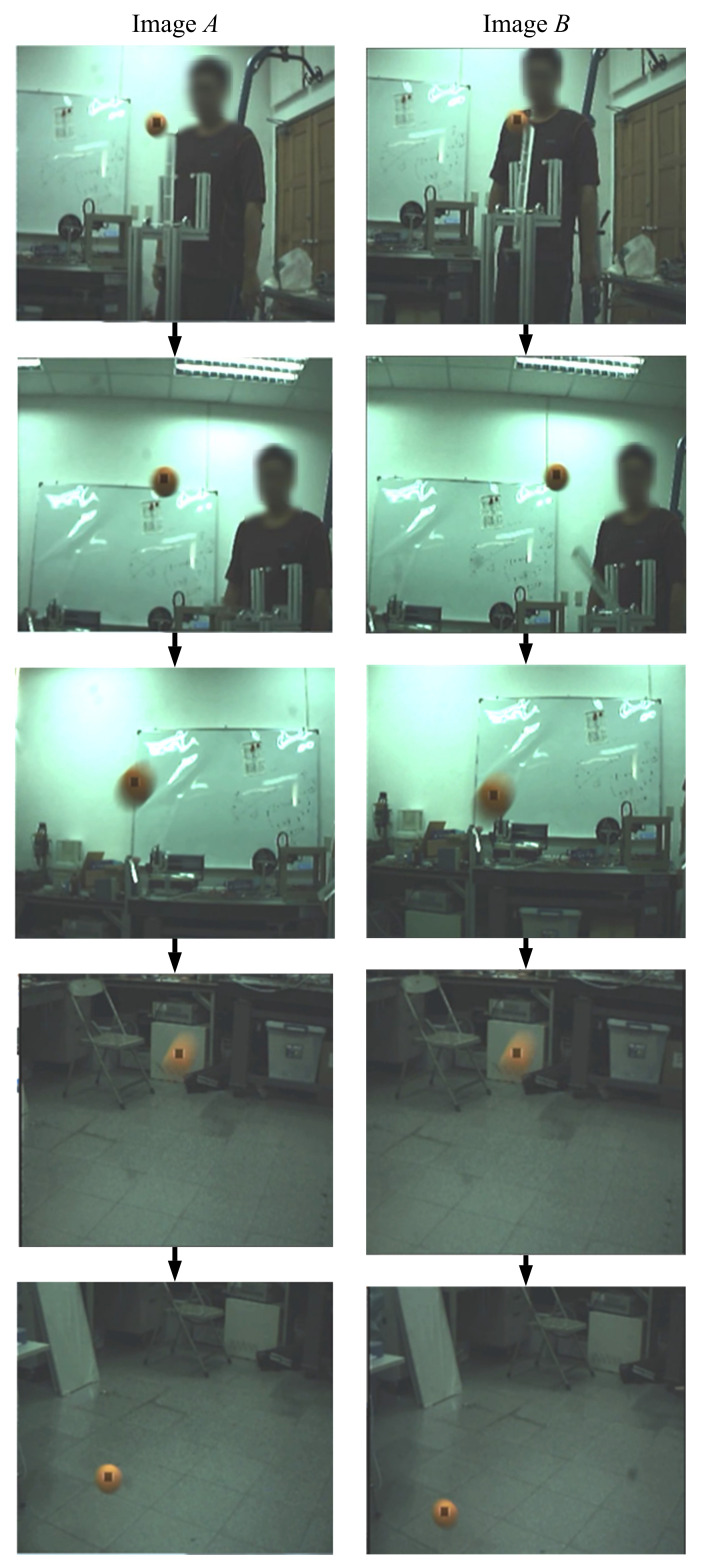
A flying ball: the sequence of images captured.

**Figure 20:**
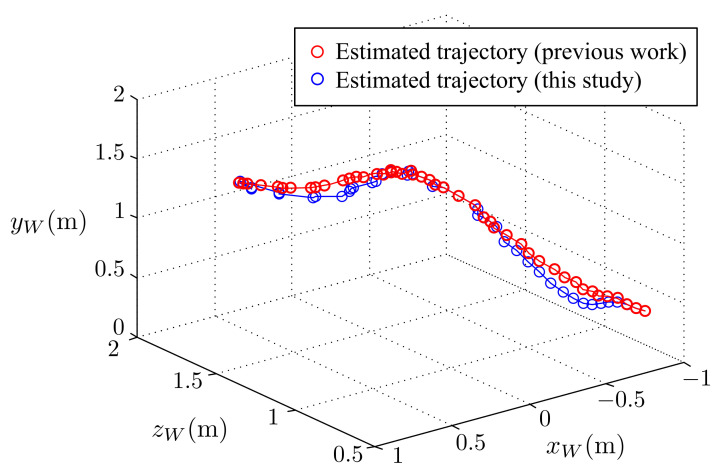
A flying ball: estimated trajectory of the ball.

**Figure 21:**
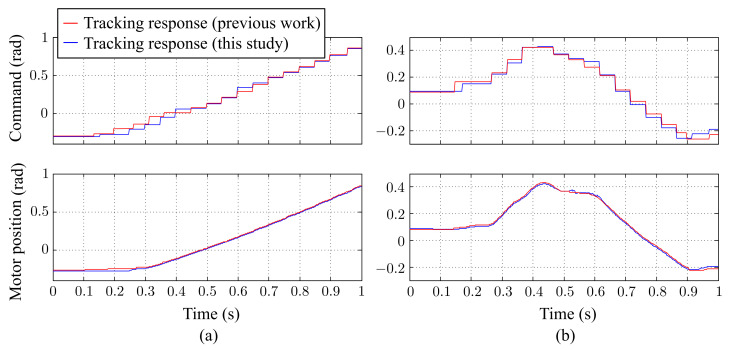
A flying ball: tracking response of (**a**) the pan-axis motor and (**b**) the tilt-axis motor.

Next, the active stereo vision camera was tested to track a flying ball. In this test, we threw the target ball at a high initial velocity. Figure 19 shows a series of images captured by the active stereo vision camera. Similar to the free-drop ball case, the visual tracking system was able to keep the ball in the FoV of the cameras. The results of a flying ball visual tracking of this study and the results of our previous work are given in Figure 20 and Figure 21.

The estimated trajectory of the ball is shown in Figure 20, and the command and time response of the pan and tilt angles are shown in Figure 21, respectively. The results are similar to those obtained for the free-drop ball case, where both of the motors were able to precisely respond to the commands. This experimental results indicate that the active stereo vision camera can track a target and keep it in the FoV even at a high initial velocity.

**Figure 22:**
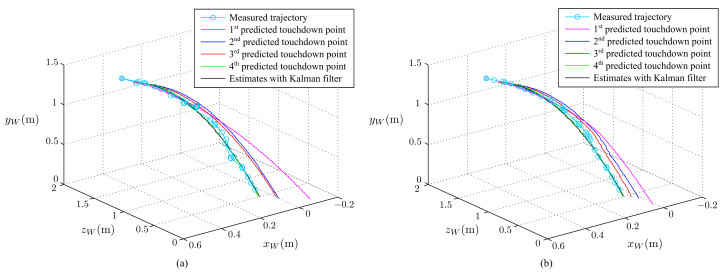
Prediction of touchdown points: (**a**) the previous work and (**b**) this study.

The effectiveness of the touchdown point prediction was investigated through experiments. In this test, the ball was thrown toward the stereo vision camera, and then the system estimated the ball’s position and velocity. With the estimated position and velocity, the future trajectory and touchdown point of the ball will be predicted by projectile motion Formulas (17)–(22). Figure 22 shows the experimental results. In this figure, the predicted trajectories converge to the measured and estimated trajectories, and the predicted and real touchdown points converge with a reasonable degree of accuracy. For comparison, the results of the touchdown point prediction of the previous work are given in Figure 22a. The results are improved.

**Figure 23:**
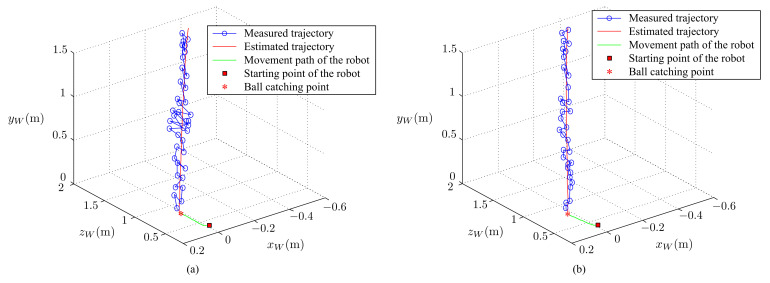
The ball’s trajectories of estimation and measurement, and the robot’s path of movement: (**a**) the previous work and (**b**) this study.

**Figure 24:**
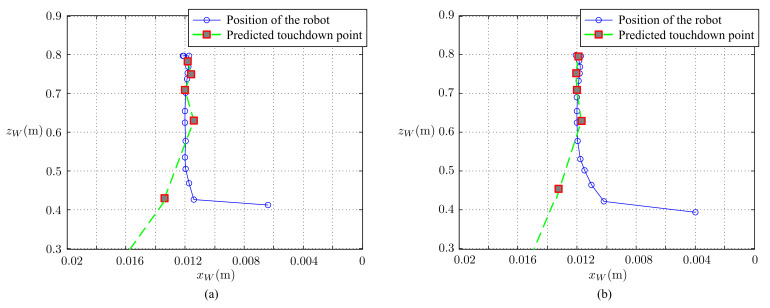
Moving path of the robot to catch the ball: (**a**) the previous work and (**b**) this study.

Finally, we tested the complete ball-catching task. In this test, the ball was thrown a 2-m distance from the mobile robot. The robot moved to the predicted point as soon as possible after receiving the command, in order to catch the ball before it touches the ground. The results of the ball-catching task of this study and the results of our previous work are given in Figure 23 and Figure 24.

In Figure 23 (3-directional view) and Figure 24 (view from the XZ plane) shown the ball’s trajectories of estimation and measurement and the robot’s path of movement. The result shows that the final predicted touchdown point and the location of the robot match very well. The residual difference between the measured trajectory and the estimated trajectory is the measurement residual. The mean value and standard deviation of the measurement residual of our previous work are 0.0529 m and 0.034 m, while those of this study are 0.0372 m and 0.019 m, respectively.

The methods proposed in this paper provided a smaller residual and variance and, therefore, more accurate measurements and more precise prediction of the touchdown point. This indicates that the robot could accomplish the ball-catching task effectively. Readers can access a link to a video clip to watch the developed system in action (https://youtu.be/En-6XcmkeBs, accessed on 30 April 2021). To determine the success rate, we performed 50 throws with random initial positions. The robot successfully caught the ball 44 times out of 50 total trials, giving an overall success rate of 88%. The success rate of our previous work was about 74%.

## 8. Concluding Remarks

In this research, a robotic ball-catching system was presented. This system consisted of multi-camera vision systems, an omni-directional wheeled mobile robot, and wireless communication. In the multi-camera vision system, the ball’s motion was tracked with an active stereo vision camera while a static vision camera navigated a mobile robot. Using a Kalman filter and the motion governing equations of a flying ball, the ball’s touchdown point was predicted with reasonable accuracy. For Kalman filtering, we found that the use of deep learning improved the accuracy and robustness.

The robot was controlled to move toward the predicted point to catch the ball before it hit the ground. The performance of the sub-systems and the proposed algorithms was verified through simulations and experiments. The results of the simulation matched the experimental results well. The experimental results confirmed that the developed robotic system combined with multi-camera vision systems could catch a flying ball.

The main contribution of this paper is to present the main issues and technical challenges of the design and system integration of a vision-based ball-catching system. Compared with the existing vision-based ball-catching systems, by combining an omni-directional wheeled mobile robot and an active stereo vision system, the ball-catching system proposed in this paper can be in a large workspace.

In future research, the image capture system used in this paper can be improved through the use of better Kalman filtering methods to restrain noise problems. Deep learning will be applied to image pre-processing, and it will be used on complex backgrounds with balls of different colors to verify the recognition accuracy. Different sensors (such as a laser range finder or RGB-D cameras) will be used for experimental comparisons. Different control laws will be applied to the omni-directional wheeled mobile robot in an attempt to increase the speed and accuracy of movement. In addition, worst-case conditions (long-distance movement or disturbance during the movement) will be applied to verify the robot’s abilities. In the simulations and experiments, different ball conditions (speed, height, etc.) and disturbances during the flight will be used to verify the system robustness.

## Data Availability

Data collected through research presented in the paper are available on request from the corresponding author.
